# Calcium Channel Blockers, More than Diuretics, Enhance Vascular Protective Effects of Angiotensin Receptor Blockers in Salt-Loaded Hypertensive Rats

**DOI:** 10.1371/journal.pone.0039162

**Published:** 2012-06-14

**Authors:** Eiichiro Yamamoto, Keiichiro Kataoka, Yi-Fei Dong, Nobutaka Koibuchi, Kensuke Toyama, Daisuke Sueta, Tetsuji Katayama, Osamu Yasuda, Hisao Ogawa, Shokei Kim-Mitsuyama

**Affiliations:** 1 Department of Pharmacology and Molecular Therapeutics, Kumamoto University Graduate School of Medical Sciences, Kumamoto, Japan; 2 Department of Cardiovascular Medicine, Kumamoto University Graduate School of Medical Sciences, Kumamoto, Japan; 3 Department of Cardiovascular Medicine, Second Affiliated Hospital, Nanchang University, Nanchang, China; 4 Department of Cardiovascular Clinical and Translational Research, Kumamoto University Hospital, Kumamoto, Japan; Osaka University Graduate School of Medicine, Japan

## Abstract

The combination therapy of an angiotensin receptor blocker (ARB) with a calcium channel blocker (CCB) or with a diuretic is favorably recommended for the treatment of hypertension. However, the difference between these two combination therapies is unclear. The present work was undertaken to examine the possible difference between the two combination therapies in vascular protection. Salt-loaded stroke-prone spontaneously hypertensive rats (SHRSP) were divided into 6 groups, and they were orally administered (1) vehicle, (2) olmesartan, an ARB, (3) azelnidipine, a CCB, (4) hydrochlorothiazide, a diuretic, (5) olmesartan combined with azelnidipine, or (6) olmesartan combined with hydrochlorothiazide. Olmesartan combined with either azelnidipine or hydrochlorothiazide ameliorated vascular endothelial dysfunction and remodeling in SHRSP more than did monotherapy with either agent. However, despite a comparable blood pressure lowering effect between the two treatments, azelnidipine enhanced the amelioration of vascular endothelial dysfunction and remodeling by olmesartan to a greater extent than did hydrochlorothiazide in salt-loaded SHRSP. The increased enhancement by azelnidipine of olmesartan-induced vascular protection than by hydrochlorothiazide was associated with a greater amelioration of vascular nicotinamide adenine dinucleotide phosphate (NADPH) oxidase activation, superoxide, mitogen-activated protein kinase activation, and with a greater activation of the Akt/endothelial nitric oxide synthase (eNOS) pathway. These results provided the first evidence that a CCB potentiates the vascular protective effects of an ARB in salt-sensitive hypertension, compared with a diuretic, and provided a novel rationale explaining the benefit of the combination therapy with an ARB and a CCB.

## Introduction

Renin-angiotensin system (RAS) blockers (angiotensin-converting enzyme inhibitors and angiotensin AT1 receptor blockers (ARB)), as well as calcium channel blockers (CCB) or diuretics, are recommended as the first line drugs for antihypertensive treatment, as indicated by Western and Japanese guidelines [1], [2]. However, these antihypertensive drugs are often used in combination in clinical practice because most hypertensive patients do not achieve their target blood pressure by monotherapy with each antihypertensive drug. Of the combination therapies, a RAS blocker combined with either a CCB or a diuretic is the main combination therapy [1], [2]. However, it is unclear which combination therapies are more effective for vascular protection.

Excess salt intake not only causes elevated blood pressure but also directly accelerates vascular injury, such as vascular endothelial dysfunction and vascular remodeling [3], [4], [5]. Furthermore, vascular injury plays a pivotal role in the development of cardiovascular events [6], [7], [8], [9]. However, it remains to be determined which combination therapy more effectively protects against vascular injury, a RAS blocker combined with a CCB or combined with a diuretic. The present study aimed to determine which combination therapy resulted in a greater suppression of salt-induced vascular injury in hypertensive rats, an ARB combined with a CCB or an ARB combined with a diuretic. We found that a CCB enhanced the improvement of vascular endothelial impairment and vascular remodeling by an ARB in salt-loaded hypertensive rats more than did a diuretic, through greater improvement of the Akt/eNOS pathway, and through greater attenuation of oxidative stress and of the mitogen-activated protein (MAP) kinase.

## Materials and Methods

### Ethics Statement

All animals experiments were approved by the Committee for Laboratory Animal Care and Use at Kumamoto University. All experimental procedures were performed in accordance with the Guidelines on Animal Experimentation that were released by the Japanese Association for Laboratory Animal Science.

### Experimental Animals

Male stroke-prone spontaneously hypertensive rats (SHRSP) and control Wistar-Kyoto rats (WKY) were purchased from Japan SLC (Shizuoka, Japan). They were fed a diet containing 8% sodium (Na) from 11 weeks of age.

### Treatment of SHRSP with Olmesartan, Azelnidipine, Hydrochlorothiazide, and their Combination

Eleven-week-old SHRSP, fed a diet containing 8% Na, were randomly assigned to 6 groups. They were orally administered (1) vehicle (0.5% carboxymethyl cellulose), (2) olmesartan (1 mg/kg/day), (3) azelnidipine (1 mg/kg/day), (4) hydrochlorothiazide (5 mg/kg/day), (5) combined olmesartan (1 mg/kg/day) and azelnidipine (1 mg/kg/day), or (6) combined olmesartan (1 mg/kg/day) and hydrochlorothiazide (5 mg/kg/day) by gastric gavage once a day for 4 weeks (from 11 to 15 weeks of age). Their blood pressure was measured by the tail-cuff method before and 1, 2, and 3 weeks after the start of drug treatment. After 4 weeks of the drug treatment, SHRSP and control age-matched WKY were perfused with phosphate-buffered saline under anesthesia with ether; the hearts, carotid artery, and aorta were rapidly excised from the SHRSP and WKY rats for measuring the various parameters described below.

### Arterial Ring Preparation and Tension Recording

After removal of the carotid artery from the SHRSP or WKY, the vessel was cut into 5 mm rings with special care to preserve the endothelium, and the rings were mounted in organ baths filled with modified Tyrode buffer (pH 7.4; NaCl 121 mmol/L, KCl 5.9 mmol/L, CaCl_2_ 2.5 mmol/L, MgCl_2_ 1.2 mmol/L, NaH_2_PO_4_ 1.2 mmol/L, NaHCO_3_ 15.5 mmol/L, and D(+)-glucose 11.5 mmol/L) aerated with 95% O_2_ and 5% CO_2_ at 37°C, as described previously [10]. The vessel rings were attached to a force transducer, and the isometric tension was recorded on a polygraph. A resting tension of 1 g was maintained throughout the experiment. The aortic rings were precontracted with L-phenylephrine (10^−7^ mol/L). After the plateau was attained, the rings were exposed to increasing concentrations of acetylcholine (Ach) (10^−9^ mol/L to 10^−4^ mol/L) or sodium nitroprusside (10^−9^ to 10^−4^ mol/L) to obtain cumulative concentration-response curves.

### Measurement of Vascular NADPH Oxidase Activity

To measure vascular NADPH oxidase activity, the excised thoracic aorta was homogenized with an Ultraturrax T8, and the supernatant was obtained by centrifugation for 5 minutes at 1,000 ×g. Aortic NADPH oxidase activities were measured by lucigenin chemiluminescence in the presence of 10 µM NADPH and 10 µM lucigenin as an electron acceptor, as described previously [11]. Chemiluminescence was recorded every 30 sec. for 15 min. with chemiluminescence reader (BLR-201; ALOKA, Japan). The chemiluminescence counts were corrected for the protein concentrations. Protein concentrations were measured by the Bradford method.

### Measurement of Vascular Superoxide

Dihydroethidium (DHE) was used to evaluate the superoxide levels in situ, as described previously [11]. The removed carotid arteries were quickly frozen, embedded in OCT, and cryostat sectioned (10-µm slices) directly onto chilled microscope slides. Sections were thawed at room temperature, rehydrated with 1× phosphate-buffered saline, and incubated for 30 minutes in the dark with the DHE (Sigma; 1 µmol/L). After washing with phosphate-buffered saline, the DHE fluorescence was visualized by fluorescent microscopy using an excitation wavelength of 520–540 nm and a rhodamine emission filter. The detector and laser settings were kept constant across all samples within individual experiments, and the control and experimental samples were always processed in parallel. DHE fluorescence was quantified using Lumina Vision version 2.2, analysis software. The mean fluorescence was quantified and expressed relative to values obtained for the WKY rats.

### Western Blot Analysis

Our detailed method has been described previously [12]. Antibodies used were as follows: anti-phospho eNOS (Ser1177) (×2000, BD Transduction Laboratories, Tokyo, Japan), anti-total eNOS (×5000, BD Transduction Laboratories, Tokyo, Japan), anti-total Akt (×2000, Cell signaling Technology Inc. Tokyo, Japan), anti-phospho Akt (×2000, Cell signaling Technology Inc. Tokyo, Japan), anti-α-tubulin (×5000, Oncogene, Tokyo, Japan), anti-phospho-ERK (×2500, Cell signaling Technology Inc. Tokyo, Japan). The intensity of the bands was quantified using NIH Image analysis software v1.61. In individual samples, each value was corrected for α-tubulin.

### Histological Examination

The hearts were fixed in 4% paraformaldehyde overnight. Then, they were embedded in paraffin, sectioned into 5-µm slices, stained with Sirius Red F3BA (0.5% in saturated aqueous picric acid, Aldrich Chemical Company) for the assessment of collagen, or immunostained with anti-ED-1 antibody (working dilution 1∶500) for the identification of monocytes/macrophages, as described [11]. The positive staining was detected using horseradish peroxidase-conjugated secondary antibodies (Nichirei, Japan), by incubating the sections with diaminobenzidine (DAKO). The number of ED-1-positive cells was counted in 4 horizontal LV in a blind manner and renal sections in individual rats; and the average of ED-1 positive cell number was obtained in individual rats [11].

### Statistical Analysis

All data are presented as the mean ± SEM. The blood pressure data at various time points after the start of drug treatment were analyzed by two-way analyses of variance (ANOVAs), and the differences between each group were determined by Fisher’s PLSD tests, using StatView for Windows (SAS Institute, Inc. Cary, U.S.A.). For other data, statistical significance was determined with one-way ANOVAs, followed by Fisher’s PLSD tests. Differences were considered statistically significant at a value of P<0.05.

## Results

### Effects on Blood Pressure of Salt-Loaded SHRSP

**Figure 1:**
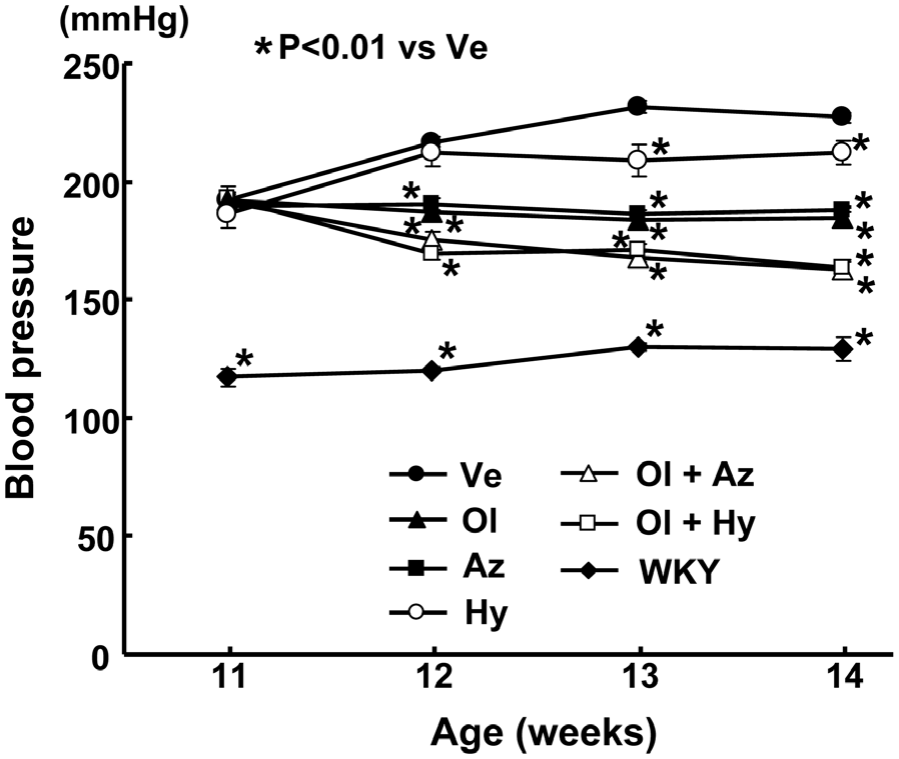
Effect on blood pressure of salt-loaded SHRSP. Abbreviations used: Ve, vehicle-treated SHRSP; Ol, olmesartan-treated SHRSP; Az, azelnidipine-treated SHRSP; Hy, hydrochlorothiazide-treated SHRSP; Ol+Az, combined olmesartan and azelnidipine-treated SHRSP; Ol+Hy, combined olmesartan and hydrochlorothiazide-treated SHRSP; WKY, Wistar-Kyoto rats. Each value represents the mean ± SEM (n = 8 in Ve, n = 7 in Ol, n = 7 in Az, n = 4 in Hy, n = 7 in Ol+Az, n = 7 in Ol+Hy, n = 8 in WKY).

As shown in Fig. 1, olmesartan or azelnidipine monotherapy significantly and similarly reduced the blood pressure of the salt-loaded SHRSP throughout the drug treatment. Monotherapy with hydrochlorothiazide significantly reduced the blood pressure of the SHRSP, although its hypotensive effect was smaller than the effect of monotherapy with olmesartan or azelnidipine. Combination therapy of olmesartan with amlodipine or with hydrochlorothiazide reduced the blood pressure of the salt-loaded SHRSP more than did monotherapy with olmesartan, azelnidipine, or hydrochlorothiazide. The addition of hydrochlorothiazide to olmesartan reduced the blood pressure of the SHRSP to a similar extent to the addition of amlodipine to olmesartan, indicating that hydrochlorothiazide added to olmesartan exerted a synergistic hypotensive effect in the salt-loaded SHRSP while azelnidipine added to olmesartan produced an additive hypotensive effect.

### Effect on Vascular Endothelial Function

**Figure 2:**
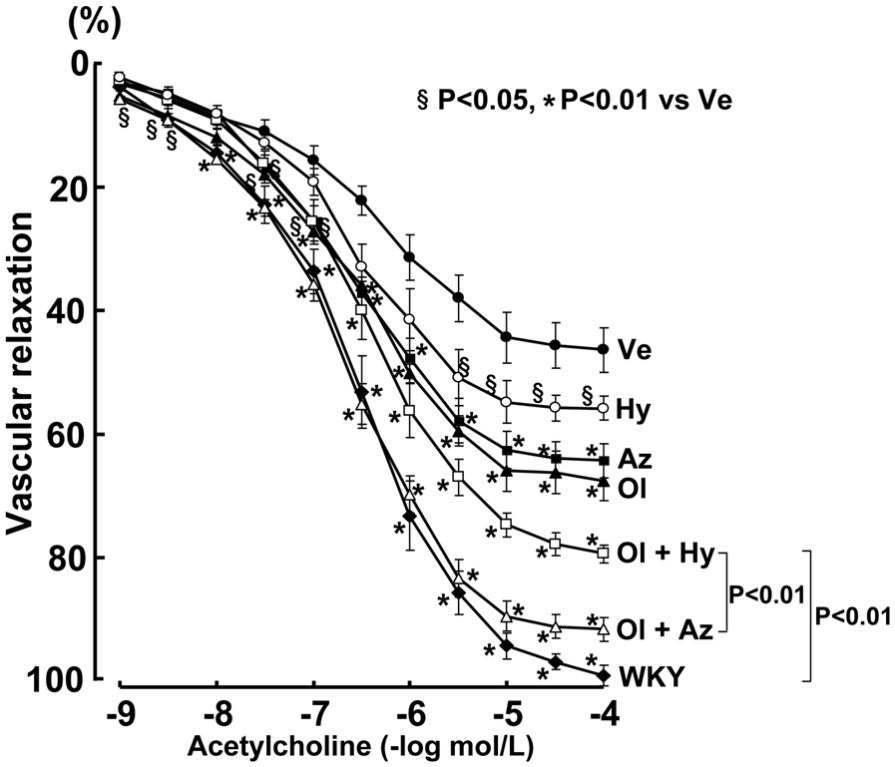
Effect on acetylcholine-induced vascular endothelium-dependent relaxation in salt-loaded SHRSP. Abbreviations used are the same as in Fig. 1. Each value represents the mean ± SEM (n = 8 in Ve, n = 7 in Ol, n = 7 in Az, n = 4 in Hy, n = 7 in Ol+Az, n = 7 in Ol+Hy, n = 8 in WKY).

As shown in Fig. 2, salt-loaded SHRSP showed markedly impaired vascular endothelium-dependent relaxation with acetylcholine compared with salt-loaded WKY rats (P<0.01). Monotherapy with all agents significantly prevented the impairment of vascular endothelium-dependent relaxation with acetylcholine. Olmesartan combined with azelnidipine and hydrochlorothiazide prevented the impairment of vascular endothelial function to a greater extent than did monotherapy with either agent. However, despite similar blood pressure measurements between the azelnidipine and hydrochlorothiazide combination groups throughout the treatment, azelnidipine enhanced the olmesartan-induced improvement of vascular endothelial function in the SHRSP to a greater extent than did hydrochlorothiazide (P<0.01).

Vascular endothelium-independent relaxation with sodium nitroprusside was comparable between the salt-loaded SHRSP and WKY rats, and all monotherapies or combination therapies did not affect it (data not shown).

### Effects on Vascular Oxidative Stress

**Figure 3:**
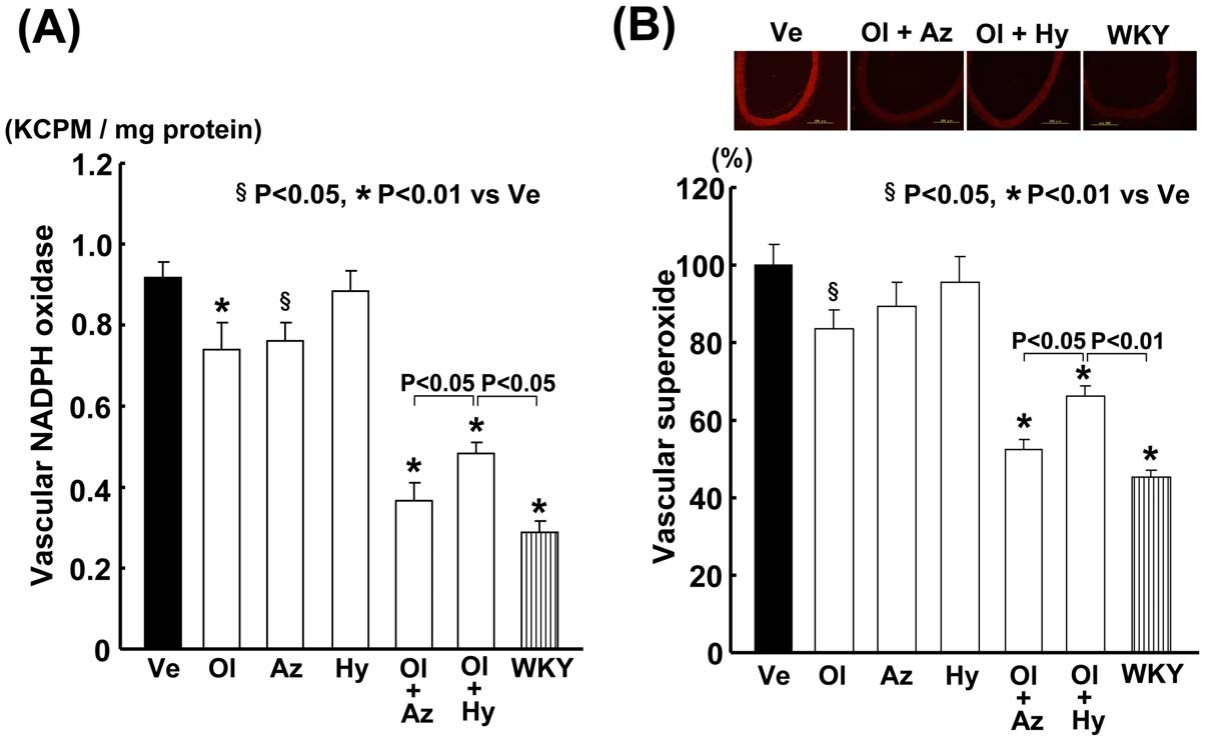
Effect on vascular NADPH oxidase activity (A) and superoxide (B) of salt-loaded SHRSP. Abbreviations used are the same as in Fig. 1. The upper panels in (B) indicate representative photomicrographs of carotid arterial DHE staining. Bar  = 200 µm. Each value represents the mean ± SEM ((A) n = 8 in Ve, n = 7 in Ol, n = 7 in Az, n = 4 in Hy, n = 7 in Ol+Az, n = 7 in Ol+Hy, n = 8 in WKY; (B) n = 12 in Ve, n = 7 in Ol, n = 7 in Az, n = 7 in Hy, n = 11 in Ol+Az, n = 11 in Ol+Hy, n = 12 in WKY).

As shown in Fig. 3, vascular NADPH oxidase activity and superoxide levels in the salt-loaded SHRSP were significantly higher than those in the salt-loaded WKY rats. Olmesartan combined with azelnidipine or with hydrochlorothiazide significantly reduced vascular NADPH oxidase activity and superoxide levels in the SHRSP, compared with any of the monotherapies. However, olmesartan with azelnidipine attenuated vascular NADPH oxidase activity (P<0.05) and superoxide levels (P<0.05) to a greater extent than did olmesartan with hydrochlorothiazide.

### Effects on Vascular eNOS and Akt

**Figure 4:**
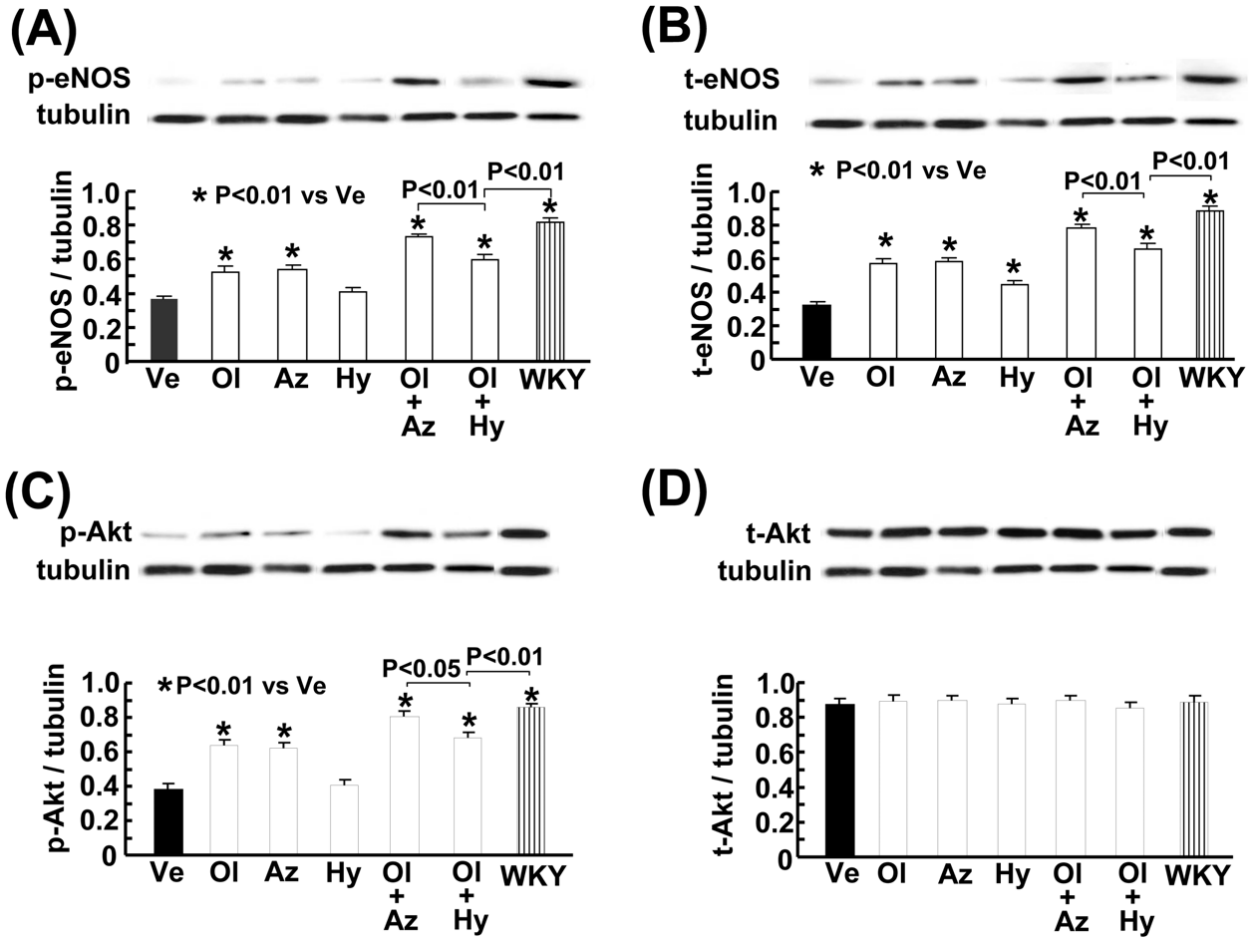
Effect on vascular phospho-eNOS (p-eNOS) (A), total eNOS (t-eNOS) (B), phospho-Akt (p-Akt) (C), and total Akt (t-Akt) (D) of salt-loaded SHRSP. Abbreviations used are the same as in Fig. 1. The upper panels in (A), (B), (C), and (D) indicate representative western blots in each group. Each value represents the mean ± SEM (n = 4 in each group).

As shown in Fig. 4 (A) and (B), the salt-loaded SHRSP showed decreased vascular phospho-eNOS and total eNOS levels, compared to the WKY rats. Olmesartan with azelnidipine or with hydrochlorothiazide significantly reversed the decrease in phospho-eNOS and total eNOS levels in the salt-loaded SHRSP. However, olmesartan combined with azelnidipine resulted in a greater reversal of the decrease in phospho-eNOS (P<0.01) and total eNOS (P<0.01) than did olmesartan with hydrochlorothiazide.

Fig. 4 (C) and (D) showed that the salt-loaded SHRSP exhibited decreased vascular phospho-Akt levels compared to the WKY rats (P<0.01). Olmesartan or azelnidipine monotherapy significantly reversed the decrease in phospho-Akt levels in the SHRSP, while hydrochlorothiazide monotherapy failed to reverse it. The combination of olmesartan with azelnidipine resulted in a greater reversal of the decrease in phospho-Akt in the salt-loaded SHRSP, compared to the combination of olmesartan with hydrochlorothiazide (P<0.05). There was no significant difference in vascular total Akt levels among the groups.

### Effects on Coronary Arterial Thickening

**Figure 5:**
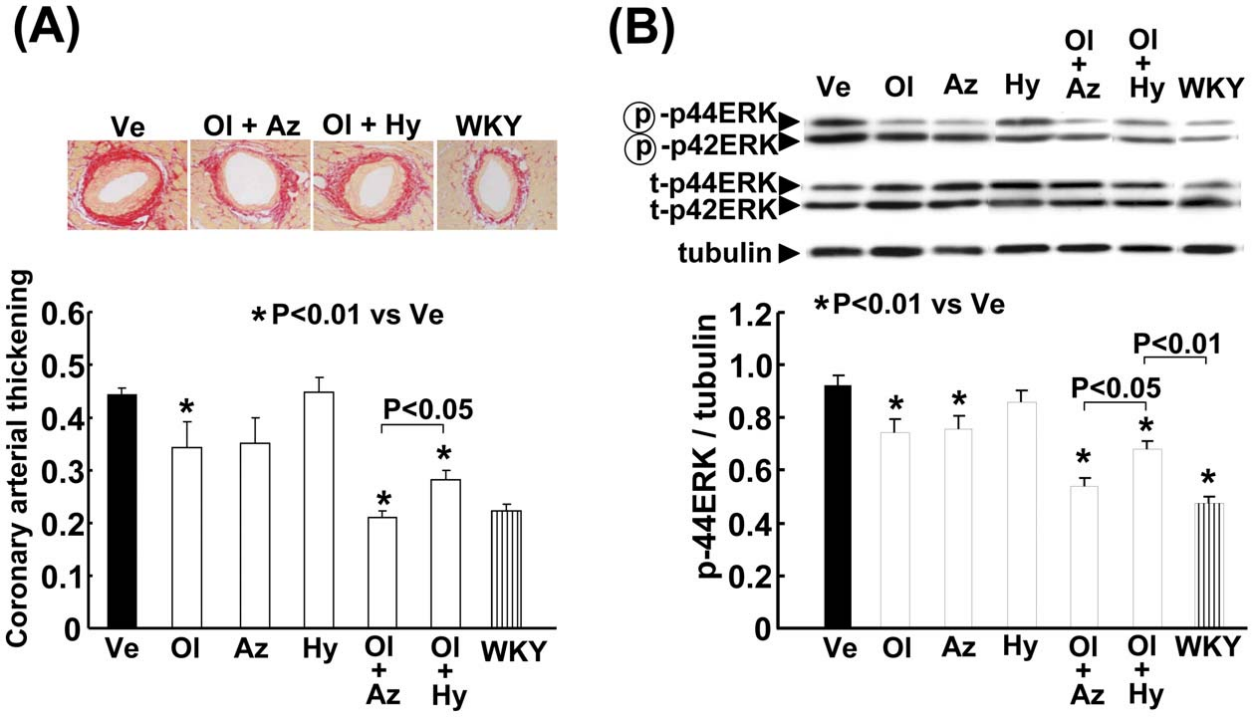
Effect on coronary arterial thickening (A) and vascular phospho-ERK (B) of salt-loaded SHRSP. Abbreviations used are the same as in Fig. 1. The upper panels in (A) indicate representative photomicrographs of the coronary artery in Sirius red-stained cardiac sections from each group. Each value represents the mean ± SEM (n = 12 in Ve, n = 7 in Ol, n = 7 in Az, n = 7 in Hy, n = 11 in Ol+Az, n = 11 in Ol+Hy, n = 12 in WKY). The upper panels in (B) indicate representative western blots in each group. Each value represents the mean ± SEM (n = 4 in each group). The value of phospho p44/42 ERK corrected for total p44/42 ERK provided the same results as the value of phospho p44/42 ERK corrected for tubulin.

As shown in Fig. 5 (A), the combination of olmesartan with azelnidipine prevented the increase in coronary arterial thickening in the SHRSP to a larger extent than did the combination of olmesartan with hydrochlorothiazide (P<0.05).

### Effects on Vascular MAP Kinase

As shown in Fig. 5 (B), vascular phospho-ERK levels were higher in the salt-loaded SHRSP than in the WKY rats (P<0.01). Olmesartan or azelnidipine alone significantly reduced the increase in vascular phospho-ERK in the SHRSP, but hydrochlorothiazide alone did not significantly affect it. The combination of olmesartan and azelnidipine resulted in a greater amelioration of the increase in vascular phospho-ERK, compared to the combination of olmesartan and hydrochlorothiazide (P<0.05).

### Effects on Cardiac Injury and Oxidative Stress

As shown in Figs. S1 and S2, monotherapy with olmesartan and azelnidipine similarly and significantly attenuated the increase in left ventricular (LV) anterior wall thickness, LV interstitial fibrosis, macrophage infiltration, NADPH oxidase activity, and superoxide in salt-loaded SHRSP, but hydrochlorothiazide alone did not significantly attenuate them. However, there was no significant difference in these parameters between the olmesartan plus azelnidipine combination group and the olmesartan plus hydrochlorothiazide group.

## Discussion

This study produced several major findings. Azelnidipine (a CCB) enhanced the amelioration of vascular endothelial dysfunction and vascular remodeling by olmesartan (an ARB) in salt-loaded hypertensive rats to a greater extent than did hydrochlorothiazide (a diuretic). The molecular mechanism underlying the enhanced improvement of vascular injury by azelnidipine, compared to hydrochlorothiazide, was associated with a greater attenuation of vascular NADPH oxidase-mediated superoxide and MAP kinase activation, and an increase in phosphorylation of Akt and eNOS by azelnidipine, compared to hydrochlorothiazide. Thus, our present work provided the molecular evidence for the superiority of a combination therapy of an ARB with a CCB over a combination of an ARB with a diuretic, and the results also provided an explanation for the clinical evidence obtained from the ACCOMPLISH trial on high-risk hypertensive patients [13].

Salt plays an important role in the progression of cardiovascular events as well as the pathogenesis of hypertension [3], [4], [5]. Epidemiologic studies showed that high sodium intake is an independent risk factor for cardiovascular disease [14]. Furthermore, cardiovascular events are reported to occur more frequently in patients with salt-sensitive hypertension compared with those with salt-insensitive hypertension [4]. Excess salt intake accelerates the development of vascular endothelial dysfunction and atherosclerosis, which are responsible for cardiovascular events and death [3], [4], [5]. Therefore, it is a clinically important issue to define an appropriate therapeutic strategy for salt-induced cardiovascular injury. Although the combination therapy of a RAS blocker with a CCB or with a diuretic is regarded as the main therapeutic strategy for hypertension [1], [2], it is unknown which therapeutic strategy is superior in preventing salt-induced vascular injury, a RAS blocker with a CCB or with a diuretic. In the current study, of note, despite comparable blood pressure lowering between the two combination therapies, a CCB (azelnidipine) enhanced the amelioration of vascular endothelial dysfunction and vascular remodeling by an ARB (olmesartan) in salt-loaded hypertensive rats, compared to a diuretic, indicating that the greater enhancement by a CCB, compared to a diuretic, of ARB-induced vascular protection was attributed to blood pressure-independent mechanisms. To elucidate the molecular mechanism responsible for superiority of a CCB over a diuretic in this study, we examined the comparative effect of a CCB and a diuretic on vascular oxidative stress, the Akt/eNOS pathway, and MAP kinase in salt-loaded hypertensive rats. Superoxide and Akt/eNOS pathway-mediated NO play a counterregulatory role in vascular endothelial function [6], [7], [8], [9]. Vascular endothelial dysfunction is mainly caused by the reduction of NO bioavailability. Superoxide, the initial product of reactive oxygen species (ROS), scavenges NO, which leads to a decrease in NO bioavailability and to the subsequent impairment of vascular endothelial function. In this work, we found that salt loading significantly impaired vascular endothelial function (Ach-induced vasodilation) in SHRSP, which was associated with increased vascular superoxide and the decreased phospho-Akt and phospho-eNOS levels. Of note, despite comparable blood pressure lowering effects between the two combination therapies, a CCB added to an ARB prevented salt-induced vascular endothelial impairment in SHRSP to a greater extent than did a diuretic added to an ARB. Furthermore, an ARB combined with a CCB ameliorated the increase in superoxide and the decrease in phospho-Akt and phospho-eNOS more potently than did an ARB combined with a diuretic. These results showed that an ARB combined with a CCB protects against salt-induced vascular endothelial impairment more than did an ARB with a diuretic, through greater amelioration of both oxidative stress and the impaired Akt/eNOS pathway. Being consistent with our previous report on Dahl salt-sensitive hypertensive rats [10], salt loading significantly decreased vascular total eNOS as well as phospho-eNOS in SHRSP. An ARB plus a CCB combination ameliorated the decrease in total-eNOS more than an ARB plus a diuretic. These results suggest that the increase in phospho-eNOS by an ARB plus a CCB combination might be partially attributed to the increase in total-eNOS by this combination therapy, although further study is needed to elucidate our assumption.

Furthermore, we also found that an ARB combined with a CCB attenuated salt-induced coronary arterial thickening more than did an ARB with a diuretic, a finding which was accompanied by a greater attenuation of vascular ERK phosphorylation by an ARB with a CCB. Taken together with the fact that ERK activation participates in the acceleration of vascular remodeling through smooth muscle cell proliferation and migration [15], [16], [17], the greater inhibition of vascular remodeling by an ARB combined with a CCB than with a diuretic seems to be at least partially mediated by a greater inhibition of ERK activation by an ARB combined with a CCB.

In conclusion, our present work provided the evidence that a CCB enhanced ARB-induced protection against salt-induced vascular injury in hypertensive rats more potently than did a diuretic. Therefore, our present experimental findings provide a novel insight into a therapeutic strategy for salt-induced vascular injury as well as an explanation for the clinical evidence obtained by the ACCOMPLISH trial [13], in which a RAS blocker combined with a CCB, compared to a RAS blocker combined with a diuretic, prevented more cardiovascular events in high-risk hypertensive patients. Furthermore, vascular endothelial dysfunction and vascular structural remodeling play a causative role in the development of hypertensive cardiovascular events. Therefore, the protective effects of an ARB plus a CCB combination against vascular endothelial dysfunction and remodeling in salt-loaded hypertensive rats seem to confer the benefit in prevention of cardiovascular events. However, extrapolation of our experimental findings to clinical situation should be made with caution.

## Supporting Information

Figure S1 Effect on left ventricular (LV) anterior wall thickness (A), LV interstitial fibrosis (B), and macrophage infiltration of salt-loaded SHRSP. Abbreviations used: Ve, vehicle-treated SHRSP; Ol, olmesartan-treated SHRSP; Az, azelnidipine-treated SHRSP; Hy, hydrochlorothiazide-treated SHRSP; Ol+Az, combined olmesartan and azelnidipine-treated SHRSP; Ol+Hy, combined olmesartan and hydrochlorothiazide-treated SHRSP; WKY, Wistar-Kyoto rats. The upper panels in (B) and (C) indicate representative photomicrographs of Sirius red-stained cardiac sections and ED-1-immunostained cardiac sections, respectively, from each group. Each value represents mean ± SEM ((A) n = 8 in Ve, n = 7 in Ol, n = 7 in Az, n = 4 in Hy, n = 7 in Ol+Az, n = 7 in Ol+Hy, n = 8 in WKY; (B) (n = 8 in Ve, n = 7 in Ol, n = 7 in Az, n = 4 in Hy, n = 7 in Ol+Az, n = 4 in Ol+Hy, n = 8 in WKY). NS, not significant.
(TIF)

Figure S2 Effect on LV NADPH oxidase activity and superoxide of salt-loaded SHRSP. Abbreviations used are the same as in Fig. S1. The upper panels in (B) indicate representative photomicrographs of cardiac DHE staining from each group. Bar = 100 µm. Each value represents the mean ± SEM (n = 8 in Ve, n = 7 in Ol, n = 7 in Az, n = 4 in Hy, n = 7 in Ol+Az, n = 7 in Ol+Hy, n = 8 in WKY).
(TIF)
