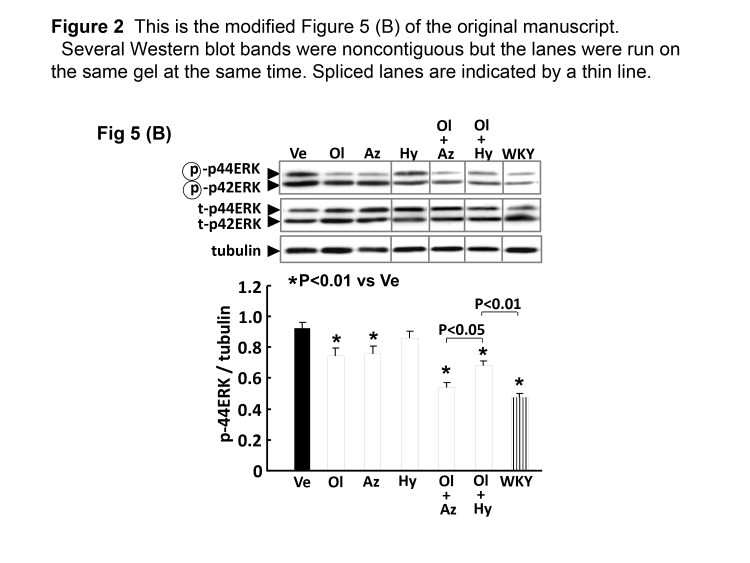# Correction: Calcium Channel Blockers, More than Diuretics, Enhance Vascular Protective Effects of Angiotensin Receptor Blockers in Salt-Loaded Hypertensive Rats

**DOI:** 10.1371/annotation/dcf5bff7-1a2e-4f9d-8ea7-cde53f04284f

**Published:** 2013-12-30

**Authors:** Eiichiro Yamamoto, Keiichiro Kataoka, Yi-Fei Dong, Nobutaka Koibuchi, Kensuke Toyama, Daisuke Sueta, Tetsuji Katayama, Osamu Yasuda, Hisao Ogawa, Shokei Kim-Mitsuyama

Representative Western blot bands of tubulin in Figure 4 (A) and (B) were placed in wrong order during preparation of the artwork. Therefore, tubulin bands in Figure 4 (A) and (B) were placed in the correct order .

The same membranes were consecutively reprobed with p-eNOS (Fig. 4 (A)), t-eNOS (Fig. 4 (B)), p-Akt (Fig. 4 (C)), t-Akt (Fig. 4 (D)), p-ERK (Fig. 5 (B)), t-ERK (Fig. 5 (B)), and tubulin. Therefore, Western blot bands of tubulin were the same among Figure 4 (A), (B), (C), and (D), and Figure 5 (B).

There is no correction in Figure 4 (C) and (D), and Figure 5 (B). However, several Western blot bands were noncontiguous and spliced together but the lanes were run on the same gel at the same time. Therefore, a thin line was added in between the spliced lanes .

Please view the corrected Figure 4 here: 

**Figure:**
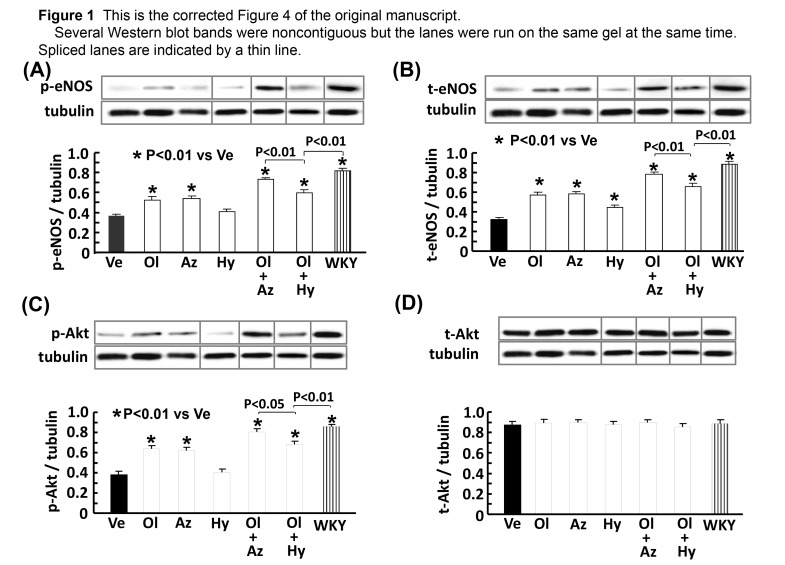


Please view the modified Figure 5B here: 

**Figure:**