# Supplementary figures and images for: Calcium Channel Blockers, More than Diuretics, Enhance Vascular Protective Effects of Angiotensin Receptor Blockers in Salt-Loaded Hypertensive Rats
Source: PLoS One. 2012 Jun 14;7(6):e39162. doi: 10.1371/journal.pone.0039162 (PMC3375277; doi:10.1371/journal.pone.0039162)

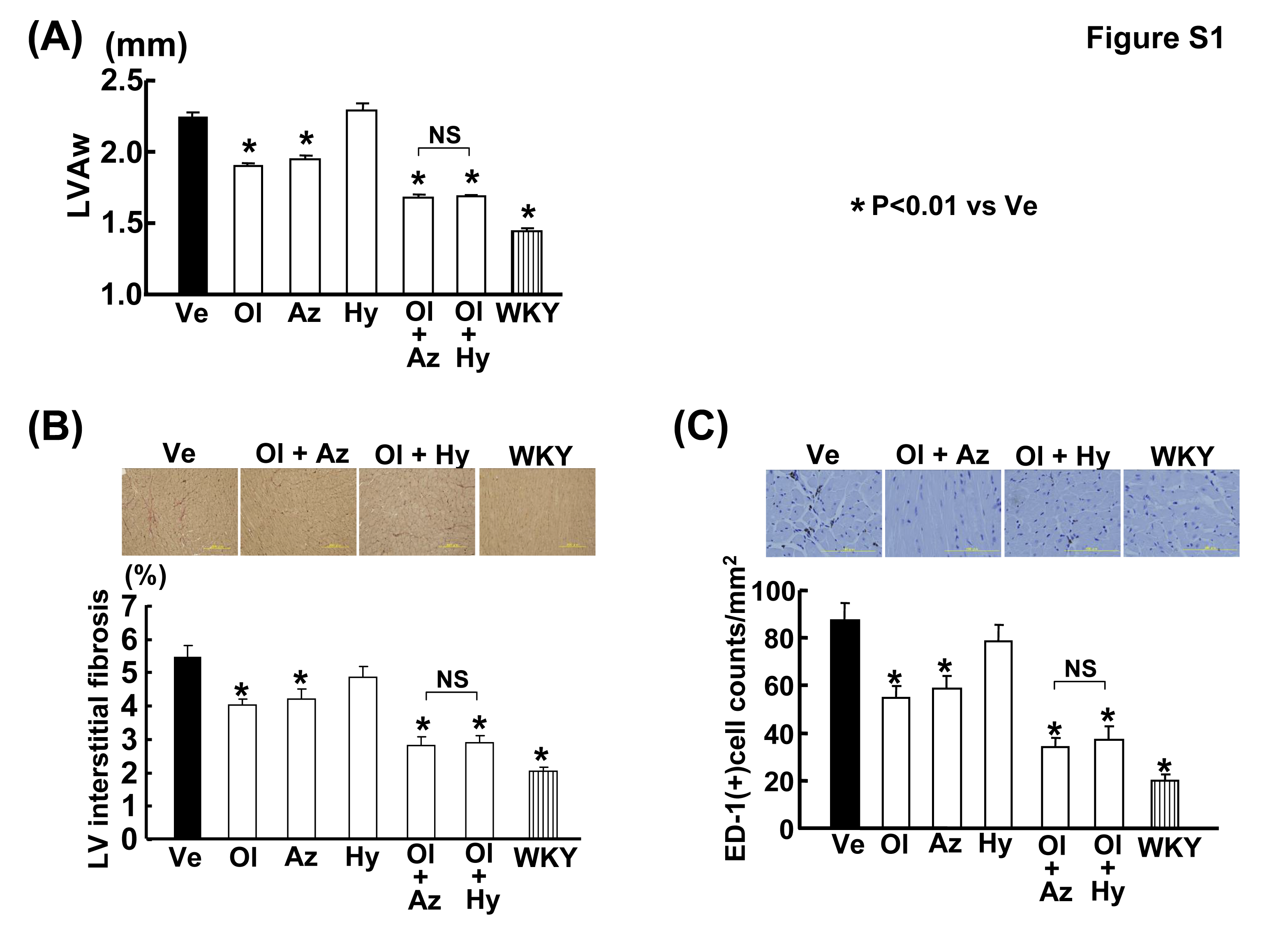

Supplement: Figure S1 — Effect on left ventricular (LV) anterior wall thickness (A), LV interstitial fibrosis (B), and macrophage infiltration of salt-loaded SHRSP. Abbreviations used: Ve, vehicle-treated SHRSP; Ol, olmesartan-treated SHRSP; Az, azelnidipine-treated SHRSP; Hy, hydrochlorothiazide-treated SHRSP; Ol+Az, combined olmesartan and azelnidipine-treated SHRSP; Ol+Hy, combined olmesartan and hydrochlorothiazide-treated SHRSP; WKY, Wistar-Kyoto rats. The upper panels in (B) and (C) indicate representative photomicrographs of Sirius red-stained cardiac sections and ED-1-immunostained cardiac sections, respectively, from each group. Each value represents mean ± SEM ((A) n = 8 in Ve, n = 7 in Ol, n = 7 in Az, n = 4 in Hy, n = 7 in Ol+Az, n = 7 in Ol+Hy, n = 8 in WKY; (B) (n = 8 in Ve, n = 7 in Ol, n = 7 in Az, n = 4 in Hy, n = 7 in Ol+Az, n = 4 in Ol+Hy, n = 8 in WKY). NS, not significant. (TIF) [file pone.0039162.s001.tif]

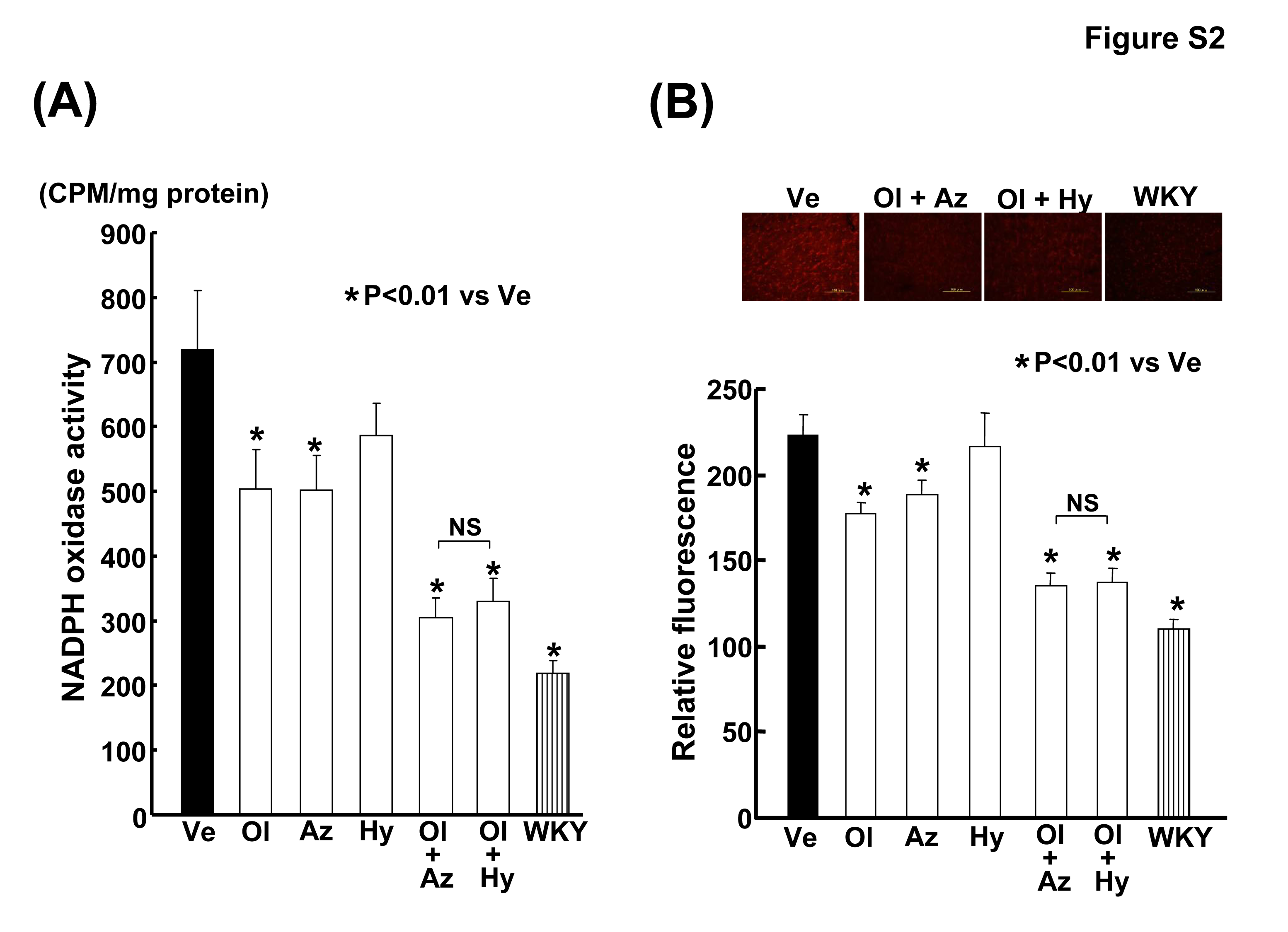

Supplement: Figure S2 — Effect on LV NADPH oxidase activity and superoxide of salt-loaded SHRSP. Abbreviations used are the same as in Fig. S1. The upper panels in (B) indicate representative photomicrographs of cardiac DHE staining from each group. Bar = 100 µm. Each value represents the mean ± SEM (n = 8 in Ve, n = 7 in Ol, n = 7 in Az, n = 4 in Hy, n = 7 in Ol+Az, n = 7 in Ol+Hy, n = 8 in WKY). (TIF) [file pone.0039162.s002.tif]
